# 

**Published:** 1828-12

**Authors:** 


### MONTHLY LIST OF MEDICAL BOOKS.

[*Medical Works cannot be entered on this List except a copy be sent for the purpose; the titles of Books having frequently been transmitted to us, as published, which have not appeared for weeks, or even months, after.*]

A Treatise on the Nature and Cure of Intestinal Worms of the Human Body. By WILLIAM RHIND, Surgeon. Illustrated by six Plates.

Remarks on the Treatment of the Insane, and the Management of Lunatic Asylums. By E. P. CHARLESWORTH, M.D.

Manual of Modern Surgery. By THOMAS CARTTIE, F.L.S. &c.

A Pocket Compendium of Anatomy, containing a correct and accurate Description of the Human Body. By EDWARD WM. TUSON.

A Treatise on Nervous Disorders. By THOMAS RICHARDS, Surgeon.

Letters on the Study and Practice of Medicine and Surgery, and on Topics connected with the Medical Profession. By JAMES WALLACE, Assistant Surgeon R.N.

Journal of Morbid Anatomy, Ophthalmic Medicine, and Pharmaceutical Analysis.

The Pupil's Introduction to Botany; containing the Description and Physiology of Plants, in explanation of the Classifications of Linnæus and Jussieu; and a copious Glossary of Botanical Terms. With Plates. By JOHN STEGGALL, M.D.

### METEOROLOGICAL JOURNAL,

### INDEX

TO THE

FIFTH VOLUME OF THE NEW SERIES

OF THE

### London Medical and Physical Journal.

PAGE

Abdomen, cases of tumors in the, from disease of the stomach . 270
———, case of punctured wound of . . . 207
Abscesses in the lungs and liver, after external injury . 269
Alkalies, action of, on iodides . 283
Ammoniac, Sal, internal use of, for contraction of the œsophagus . . . . . 175
Ammonia, decomposition of, by metals . . . 367
Anasarca cured by tartar-emetic ointment . . . 74
Anatomical Committee, report of, 373
Anatomy, Elements of, Descriptive and Practical . . 462
Aneurism, gluteal, successful case of . . . . 30
Angina maligna successfully treated by nitrate of silver . 558
Ani, Sphincter, on painful and permanent spasmodic stricture of . . . . 481
Anus, artificial, M. Dupuytren on 210
———, case of . . 238
———, proposal to make an, in schirrous rectum . 567
———, arising from ulceration of the colon, which, after discharging feces for 81 days, spontaneously closed . 110
———, cases of constriction of, without fissure . . 489
———, ditto, with fissure . 485
Apoplexy, observations on the cause of . . . 136
Apothecaries, regulation of the Court of Examiners of . 572
———, regulations for the examination of . . 475

PAGE

Arsenical solution, use of in chronic headache . . . 173
Arteries, spontaneous obstruction of the canals of . . 150
———, chronic inflammation of 86

Bladder, case of rupture of . 233
——— enlarged . 466
Bleeding, inflammation of vein and death from, with purulent depôts in the shoulder and knee 522
Blue colour by action of muriatic acid upon albumen . . 366
Bones, utility of pressure in the reunion of fractured . . 494
Brain, diminution of, from wasting of the body . . 396
———, curious affection of, in four brothers and sisters . . 169
———, loss of, from injury . 281
———, Mr. Brodie on injuries of 348
———, hypertrophia of . . 463
Breast, fungus hæmatodes, case of, 520
———, hydatids in the female, resembling a schirrous tumor 169
Breasts, case of sanguineous discharge from . . . 362
Bronchitis, chronic, treated by external use of acetate of morphia . . . 88
Brookes' (Mr.) Museum. sale of 177
Burns, treated with chloruret of lime, and of sodium . . 564

Calculus, case of urinary, in the glans penis . . . 365
Cambridge, university of, examination in the, for M.B. degree 178, 285, 371
Cancers, extirpation of, in rectum and vagina . . 472

PAGE
Cancer of the womb, relieved by acetate of ammonia . . 556
———, pus never found in . 36
Cantharides, case of poisoning by 50
Catalepsy, case of, with mania 315
Cerebro-spinal Fluid, memoir on 394
Cherrattah, on its deobstruent and stomachic properties . 493
Chlorine in chronic affections of the lungs . . . 173
Cholera, on Epidemic, of India 166
Chorea, use of gymnastic exercises in . . . 89
Choroid plexus, encysted dropsical tumors in . . 67
Circulation, disorders of, in insanity 534
Citric acid from gooseberries . 366
Cold affusions in external inflammations, utility of . . 122
Colon, misplaced, with enteritis 234
Compression of the brain . 351
Conjunctiva, on diseases of the tunica . . . 331
Contagion, specific, generated in hospital gangrene . . 145
Convulsions cured by ligature . 470
Copaiva, Balsam of, producing eruptive disease . . 275
Cornea, opacity of, cured by insufflations of calomel . 564
Corporeal Organization, on the alliance between it, the animal life, and the life of the understanding . . . 412
Corydalin, a new vegetable alkali, description of . . 367
Cough, obstinate, from elongation of uvula . . . 27
Cow-pox from grease in the horse 168
Cranium, fracture of, with extensive depression . . 518
———————, with abscesses in the liver and lungs . 435

Deafness, on the causes, prevention, and cure of . . 339
——— cured by electricity . 174
Demonomania, case of . . 254
Depression of the cranium, case of extensive, with fracture . 518
Diarrhœa, periodical, producing tendency to gout . . 449
———, chronic, nux vomica in 560
Dysentery, cases of chronic . 516
———, epidemic, on the, of Dublin, in 1825 . . 83
———, cases of, in which the hydrochloruret of lime was used 41
Dysmenorrhœa, relieved by acetate of ammonia . . 556

PAGE
Electricity, deafness cured by 174
Emphysema following labour . 474
Epidemic at Paris . . 557
Epilepsy, stramonium in . 90
Ergot of Rye, on the use of . 159
Erysipelas, cases of . . 126
———, clinical remarks on treatment of, by Mr. Guthrie 128
———, different modes of treating . . . 566

Examination for M. B. degree at Cambridge . 178, 285, 371
Eye, Compendium of Diseases of 327
——, chronic and acute inflammation of, treated by lunar caustic ointment and the oxymuriate of mercury . . 193
Eyes of a child, words in the . 285

Face, paralysis of, with trismus 514
Femur, congenital disease of, illustrating the pathology of interstitial absorption of the cervix femoris . . 149
Fœtation, extra-uterine, in which the fœtus remained in the abdomen forty years . . 133
Fever, cases of, treated by hydrochloruret of lime . . 38
———, remarks on . . 295
Foramen Ovale, case of open 43, 290
Fractures, treatment of ununited 317
———, remarks on long standing . . . . 553
Frontal bone, tumor over the orbitary plate of . . 520
Fungus hæmatodes of the breast, case of . . . ib.

Gall, Dr. account of his disease 569
———, biography of . 477
Gangrene, on hospital, in the army in the Peninsula . 141
——— of the thigh, with the question of amputation . 227
Gas, on inspiration of inflammable 362
Gonorrhœa, followed by phagedæna, hemorrhage, and eruptions . . . . 507
Gout, case of . . . 171
——, causes of . . . 451
——, analysis of Mr. Rennie's Treatise on . . . 441

Harrison, Dr., defendant in an action brought by the College of Physicians . . 179
Headache, chronic, cured by Fowler's arsenical solution . 173

PAGE

Head, injury of, with loss of brain . . . . . 281
Heart, wound of, patient surviving ten days . . . 526
——, hypertrophy of, with ossification . . . 560
——, organic disease of, causing sudden death . . 135
Hellebore in hydrophobia . . 2
Hemorrhage, hereditary, remarks on . . . 308, 404
Hemorrhoids, internal, danger of hemorrhage after extirpation 471
Hernia, case of strangulated scrotal, removal of portion of omentum . . . 433
——, case of strangulated inguinal . . . 434
——, use of tobacco in . 114
Herpes Phlyctenoides proved not to be contagious . . 556
—— cured by diet and mercurials . . . . . 174
Hip, dislocation of, case . 519
Hip-joint, spontaneous dislocation of . . . . 286
Hæmoptysis, remarks on . 170
——, very large quantity of blood lost in . . 15
Human race, on the species or varieties in the . . 368
Hydatids, observations on . 99
——, extraordinary circumstance connected with the history of . . . . 67
—— of the liver, operation for . . . . . 437
Hydrocele, attempt to cure by insertion of solution of nitrate of potash . . . . 470
Hydrocephalus acutus, vomiting in, produced by noise . 235
——, acute, case of, cured by spontaneous abscess 465
——, with enlargement of the skull . . . 73
——, Dr. Monro on . 66
Hydrophobia treated by hellebore 2
——, observations on 1, 5, 8

Iliac artery, ligature of external, successful case . . 471
——, internal, successful case of ligature of, for gluteal aneurism . . . 30
Inflammation, chronic, in various structures, treatment of . 57
——, on the Proximate Cause of . . . 403

PAGE

Inflammations, external, cured by cold affusions . . 122
Insanity, on the forms, causes, symptoms, and treatment, moral and medical, of . 455, 528
——, pathological examination of cases of . . . 530
——, Dr. Burrows' division of 541
Intermittent fever, use of piperine in . . . . . 117
Intestinal hemorrhage, nux vomica in . . . . . 560
Iodine successfully used in indurated enlargement of uterus . 49

Joints, on Diseases of, Mr. Scott 57
——, cases of chronic affections of . . . . . 319

Kidneys, on hypertrophy of . 363
——, lobulated . . ib.
——, anatomy and diseases of ib.
——, on atrophy of . 364
Kidney, fatal case of disease of 299
Knee-joint, on removal of loose substances from . . 324

Larynx, suffocation caused by a leech in the . . . 168
——, needle in the, case of . 525
Lawrence, Mr. on his election into the council of the College of Surgeons . . . 373
Lead, superacetate of, hydrophobia treated by . . 5
Lime, hydrochloruret of, used in dysentery . . . 41
—— in fever . ib.
Liver, abscess of, in a case of fractured cranium . . 435
——, hydatids of . . 437
——, cured by operation . . . . . 565
——, case of gun-shot wound of 233
——, on one of the functions performed by, in the fœtus, and in amphibious animals . . 202
Lung, hepatised, case of . 557
Lungs, abscesses of, in a case of fractured cranium . . 435
——, on the physical signs of the diseases of the . . 258

M'Gregor, Sir P., death of, announced . . . 177
Mammæ, on affections of, liable to be mistaken for cancer . 32
Maxillary bone, destruction of, case 466
Mental disease, cases of . 250

PAGE
Midwifery, Report of Cases in the London and Southwark Midwifery Institution . 18
Moles, vision of . . 555
Morphia, acetate of, external application of, in chronic bronchitis . . . 88

Necrosis, case of, of the maxillary bone, with regeneration of the teeth . . . . 466
Neck, extirpation of very large steatomatous tumor from . 46
Nux vomica in chronic diarrhœa and intestinal hemorrhage . 560
Nymphomania, from inflammation of cervix uteri . . 474

Œsophagus, contraction of, relieved by internal use of sal ammoniac . . . 175
Omentum, removal of a portion of 433
Onychia maligna, different modes of curing . . . 562
Opium in insanity, observations on the use of . . . 256
———, detection of small quantities of . . . 282
Orbit, tumor in, case of . 56
Organised Bodies, on Changes of Structure in . . . 386

Painter's colic, on paralysis in 297
Paralysis of the face, case of, with trismus . . . 514
Parturition, on difficult cases of 159
Pathology, observations on some points of . . . 87
Penis, case of urinary calculus in the glans . . . 365
——, cancerous, amputation of, unnecessary . . . 473
Perforation in the stomach, cases of . . . . . 302
Phthisis, smoking of belladonna in 557
Physicians, College of, meetings at 92
————————, power of the 191
————————, v. Harrison 179
Piperine, observations on, in intermittent fever . . 117
Pleura, on the physical signs of the diseases of the . . 258
Pneumo-gastric nerves, influence of . . . . . 84
Poisons absorbed by wounds in the skin, treatment of . 568
Poisoned wounds, cupping glasses to . . . . . 283
Poisoning, case of, by cantharides 51

PAGE
Pubis, Symphisis, separation of the, in a case of rupture of the bladder . . . 232
Pulmonary artery, cases of contraction of . . . 130
Pupil, on artificial . . 337
Pupils, number of, at anatomical schools . . . 372
Pus, depositions of, occurring in the lungs and other viscera, after injuries of different parts of the body . . . 267

Quinine, sulphate of, use of, in fevers . . . 172

Recruits, Hints for the Examination of . . . 151
Rectum, extirpation of cancers in 472
Religion in reference to insanity 459
Report of the Anatomical Committee . . . 373
Retina, on inflammation of . 335
Rheumatismus febrilis, remarks on 21
Ribs, successful cases of resection of . . . 491
Rumination in a man, case of . 361
Rye, ergot of, on the use of . 159

Salivation, from the external use of tartar emetic . . 274
Sanguineous discharge from the breasts . . . 362
Sexes, proportion of the . 368
Simarouba, use of, as an antispasmodic . . . 83
Small-pox, pustules opened by nitrate of silver . . 555
Sodium, chloruret of the oxide of, its good effects upon ulcers 466
Soldiers, feigned disabilities of 151
Spina bifida, remarks on . 397
Stethoscope, cases to illustrate the utility of . . . 75
Stomach, cases of perforation in the . . 302, 422, 555
————, case of rupture of, produced by vomiting . . 265
————, termination of in a cul-de-sac . . . 84
Stone, bilateral operation for . 511
——, new operations proposed for the . . . 469
——, Neapolitan operation for the . . . . 564
Stramonium, Datura, practical observations on . . 89
Structure, changes of, in organised bodies . . 97, 386

PAGE
Surgeons, College of, regulations respecting reading-room . 370
Syphilis, natural or spontaneous cure of . . . 149

Tænia, essential oil of semen santonicum for . . . 558
Teeth, description of mineral . 286
———, regeneration of, case . 466
Theology, Natural, Mr. Paxton's edition of Paley on . . 287
Thigh, gangrene of . . 227
Thigh-bone, on Fractures of, analysis of Mr. Amesbury's work 544
Thyroid gland, disease of the . 401
Tobacco, use of, in hernia . 114
Tonsils, removal of . . 175
Tongue, cancer of, removed by incision and ligature . 474
Tracheotomy in croup . . 568
Transactions of the Association of the Fellows and Licentiates of the King and Queen's College of Physicians in Ireland 74
Trismus, case of, with paralysis of the face . . . 514
Tubercles, on the origin of . 150
Tumors, cases of, removed from different parts . . 53
———, on Sanguineous, of an equivocal character, which appear to be aneurisms of the arteries of the bones . 280
———, removal of, from different parts . . . 138

Ulcers, treatment of, by plates of lead . . 279, 562
University, London, lectures at the 92
Ureters, anatomy and diseases of 363
Urethra, a Series of Observations on Strictures of, with an account of a new method of treatment . . . 240

PAGE
Urethra, strictures of, description of a new instrument for the division of . . 246
Uteri, polypus, fatal case of ligature to . . . 474
Uterine hemorrhage, utility of friction in . . . 19
Uterus wanting, case of . 463
———, prolapsus of, case of . 427
———, polypus of, case of . 428
———, case of chronic inflammation of the neck of . ib.
———, rupture of, at the time of quickening . . . 430
———, extirpation of 275, 430, 470
———, case of tumor in the . 235
———, indurated enlargement of, successfully treated with iodine . . . 49
Uvula, portion of, removed in a case of obstinate cough . 27

Vagina, closure of, after difficult labour . • . 513
———, extirpation of cancers in 472
Vein, inflammation of, with purulent depôts in shoulder and knee . . . . 522
Veins, Inflammation of, abstract of Mr. Arnott's paper on . 573
Viscera, transposition of . 361
Vision, anomalous . . 168
Vomiting in hydrocephalus acutus, produced by noise . 235

Water, supply of, to the metropolis . . . . 93
White swelling cured by iodine frictions . . . 564

Yellow fever, case of disease at Portsmouth resembling . 105

---

COLLECTANEA . . *Pages* 85, 168, 265, 361, 463, 555
INTELLIGENCE . . . 91, 177, 285, 369, 475, 571
METEOROLOGICAL REGISTER 96, 192, 288, 384, 480, 576

### CRITICAL ANALYSES.

PAGE
Surgical Observations on the Treatment of Chronic Inflammation in various Structures, particularly as exemplified in Diseases of the Joints. By JOHN SCOTT, Esq. . . . . . . . 57
The Morbid Anatomy of the Brain. By Dr. MONRO . . . 66
Transactions of the Association of the Fellows and Licentiates of the King and Queen's College of Physicians in Ireland . . . 74
Sketch of a Medical Report on the Epidemic Dysentery which prevailed in Dublin in 1825. By Dr. O'BRIEN . . . . 83
Clinical Observations made during the Epidemic Fever of 1826. By Dr. REID . . . . . . . . . . ib.
Transactions of the Medico-Chirurgical Society of Edinburgh, Vol. III. Part I. . . . . . . . . . . 141
Hints to young Medical Officers of the Army on the Examination of Recruits, and respecting the feigned Disabilities of Soldiers, &c. By Mr. MARSHALL . . . . . . . 151
On Difficult Cases of Parturition, and the use of Ergot of Rye. By Mr. MICHELL . . . . . . . . . 159
Notes on the Epidemic Cholera. By Dr. KENNEDY . . . 166
A Series of Observations on Strictures of the Urethra, with an Account of a new Method of Treatment. By Mr. STAFFORD . . 240
Cases of Mental Disease, with Practical Observations on the Medical Treatment. By Dr. MORISON . . . . . . 250
A Rational Exposition of the Physical Signs of the Diseases of the Lungs and Pleura. By Dr. WILLIAMS . . . . . . 258
A Compendium of the Diseases of the Human Eye, &c. By Mr. WATSON 327
Deafness; its Causes, Prevention, and Cure. By Mr. STEVENSON . 338
Pathological and Surgical Observations relating to Injuries of the Brain. By Mr. BRODIE. Part I. . . . . . . . . 348
A Treatise on Gout, Apoplexy, Paralysis, and Disorders of the Nervous System. By Mr. RENNIE . . . . . . . 440
Commentaries on the Causes, Forms, Symptoms, and Treatment, Moral and Medical, of Insanity. By Dr. BURROWS . . 455, 528
Elements of Descriptive and Practical Anatomy, for the use of Students. By Mr. QUAIN . . . . . . . . . 462
Observations on the Nature and Treatment of Fractures of the upper third of the Thigh-bone, &c. By Mr. AMESBURY . . 544

---

### NAMES OF AUTHORS,

*Of whose Works, Observations, &c. either a detailed Account, or more or less general View, is given in the present Volume.*

ADAMS, Mr. FREDERICK, on Hydrophobia . . . . 1
ALISON, Dr. on the Origin of Tubercles . . . . 150
AMESBURY, Mr. analysis of his work on Fractures of the Thigh-bone 544
ANDERSON, Dr. his case of Artificial Anus . . . . 238
ARNOTT, Mr. abstract of his paper on Inflammation of the Veins . 573
ANEBILL, Mr. on the Removal of loose Substances from the Knee-joint 324
BAKER, Mr. on the Herb Cherrattah as a Deobstruent, &c. . . 493
BALLINGHALL, Dr. his cases of Tumors removed from different parts 53, 138
——— on Gangrene of the Thigh, with the question of amputation 227
——— his cases of Chronic Affections of the Joints . . 319
——— Ununited Fractures . . . . 317

PAGE
BANNER, Mr. J. M. his case of Extirpation of the Uterus . . 430
——————— Strangulated Inguinal Hernia . . 434
BARON, Dr. on Changes of Structure in Organised Bodies . 97, 387
BELL, Mr. C. his case of Trismus, with Paralysis of the Face . . 514
BLAKE, Dr. his case of Foramen Ovale found unclosed . . 290
BLANDIN, M. his case of Extraction of a Needle from the Larynx . 525
BLUNDELL, Dr. his case of Extirpation of the Uterus . . . 275
BOGGIE, Dr. on Hospital Gangrene . . . . . . 141
BOUILLAUD, Mr. on the Diseases of the Kidneys and Ureters . . 363
BOW, Dr. his observations on Fever . . . . . . 295
——— on the Proximate Cause of Inflammation . . . 403
BRESCHET, M. his case of Uterus wanting . . . . . 463
——————— on Sanguineous Tumors of an equivocal character . 280
BRODIE, Mr. his Observations relating to Injuries of the Brain . 348
BRYANT, Mr. his case of Disease of the Kidney . . . 299
BURROWS, Dr. Commentaries on Insanity, analysis of . 455, 528
CANHAM, Dr. his case of Maniacal Catalepsy . . . . 314
CARPENTER, Mr. on the use of Piperine in Intermittent Fevers . 117
CHEVALIER, Mr. T. on Hæmoptysis . . . . . . 15
CITTADINI, M. his successful cases of Resection of the Ribs . . 491
COATES, Dr. his observations on Hereditary Hemorrhages . 312, 404
CRAMPTON, Dr. (Dublin,) his case of open Foramen Ovale . . 43
DALY, Dr. on Extirpation of a large Steatomatous Tumor from the Neck 46
DANCE, M. on Hypertrophy of the Brain . . . . . 463
DAVIE, Mr. his case of Gonorrhœa followed by Eruptions . . 507
DAVIS, Dr. CHARLES, on the use of Bleeding in Hydrophobia . 9
DUPUYTREN, M. his attempt to cure Hydrocele by Nitrate of Potash 470
———————, on the Danger from Hemorrhage after the Extirpation of internal Hemorrhoids . . . . . . . . 471
———————, Memoir on Artificial Anus . . . . . 210
EBERMAIER, Dr. his cases of Perforation in the Stomach . 302, 422
ELSE, Mr. his case of Rupture of the Uterus at the time of quickening 430
ESTLIN, Mr. his case of Enlarged Bladder . . . . . 466
FIELD, Dr. his case of Punctured Wound of the Abdomen . . 207
FLETCHER, Mr. Amputation of a large Fungus Hæmatodes of the Breast 520
GIACOMO CARDONE, Signior, on Inspiration of Inflammable Gas . 362
GUTHRIE, Mr. his cases of Chronic and Acute Inflammation of the Eye 193
——————————— Erysipelas . . . . . 126
HALL, Mr. his case of Artificial Anus . . . . . . 110
HARE, Dr. on detecting the Presence of Opium in very small quantities 282
HEISKELL, Dr. his case of Extra-uterine Fœtation, &c. . . 133
HORNER, Dr. Observations on Pathology . . . . . 87
HOSACK, Dr. on Removal of Tonsils . . . . . . 175
IVES, Dr. (New York,) his case of Poisoning by Cantharides . . 50
JACOBSON, Dr. his case of Sanguineous Discharge from the Breast . 362
JENKINS, Mr. on Hydrophobia . . . . . . . 5
JENNINGS, Mr. E. A. on one of the Functions performed by the Liver, &c. 202
KEATE, Mr. his case of Fracture of the Cranium, &c . . . 435
———————— Closure of the Vagina after Labour . . 513
KENNEDY, Dr. on Epidemic Cholera of India . . . . 166
KITSON, Mr. his observations on the Cause of Paralysis in Painter's Colic 297
KNOX, Dr. on Disease of the Femur . . . . . 149
KRIMER, Dr. his case of Necrosis, with destruction of Maxillary Bone 466
LARREY, M. A. his case of Hydrocephalus Acutus . . . 465
LISFRANC, Mr. on Extirpation of Cancers within the Rectum and Vagina 472
——————— on the unnecessary Amputation of a Cancerous Penis . 473
——————— his remarks on Nymphomania . . . . 474
MACLACHLAN, Dr. on Fracture of Cranium and extensive Depression 518
MAGENDIE, M. Physiological Memoir on the Cerebro-spinal Fluid . 394
MARJOLIN, M. his fatal case of Ligature to Polypus Uteri . . 474

PAGE
MARSHALL, Mr. on the Examination of Recruits, &c. . . . 151
MAYO, Mr. his case of Strangulated Scrotal Hernia . . . 434
MENON, Dr. on the Treatment of Ulcers by Plates of Lead . . 279
MESTIVIER, M. his case of Gout . . . . . . 171
MICHELL, Dr. (Pennsylvania,) his cases of Chronic Dysentery . 516
MICHELL, Mr. on Difficult Cases of Parturition, &c. analysis of . 159
MONRO, Dr. analysis of his work on Morbid Anatomy of the Brain . 66
MORRISON, Dr. cases of Mental Disease . . . . . 250
NIELL, J. Esq. his case of Disease resembling Yellow Fever . . 105
OTTO, Dr. on chronic Headache cured by Fowler's arsenical solution . 173
PAGENSTECHER, M. his case of Contracted Œsophagus, &c. . . 175
PARRISH, Dr. (Pennsylvania,) on Affections of the Mammæ liable to be mistaken for Cancer . . . . . . . . 32
PAXTON, Mr. his edition of PALEY's Natural Theology . . 287
PENCOCK, Dr. on applying Cupping Glasses to Poisonous Wounds . 283
PHYSICK, Dr. on Obstinate Cough . . . . . . 27
QUAIN, Mr. analysis of his Elements of Descriptive and Practical Anatomy 462
RECAMIER, M. on Hydatids of the Liver . . . . . 437
REID, Dr. (Dublin,) on Fever and Dysentery, in which the Hydrochloruret of Lime was employed . . . . . . 38,41
RENNIE, Mr. analysis of his Treatise on Gout, Apoplexy, Paralysis, &c. 440
RICHERAND, M. his successful case of Ligature of external Iliac Artery 471
ROSE, Mr. on Deposition of Pus and Lymph in the Lungs and other Viscera 267
SAVART, M. on Decomposition of Ammonia by Metals . . . 367
SCHWARTS, Dr. his case of Urinary Calculus lodged in the Glans Penis 365
SCOTT, Mr. analysis of his Treatise on Diseases of the Joints . . 57
SELWYN, Mr. his case of Disease of the Thyroid Gland . . 401
SEYMOUR, Dr. cases of Tumors in the Abdomen, arising from Organic Disease of the Stomach . . . . . . . 270
SEWALL, Dr. his cases of Injury of the Head, with a loss of Brain . 281
SHEPPARD, Mr. his case of Dislocation of Hip . . . . 519
SQUAER, Mr. on Rheumatismus Febrilis . . . . . 21
STAFFORD, Mr. analysis of his work on Strictures of the Urethra . 240
STEVENSON, Mr. on Deafness, its Causes, Prevention, and Cure . 339
THOMAS, Mr. his Metaphysico-Physiological Essay upon the Nature of the triple Alliance of Corporeal Organization, Animal Life, and the Life of the Understanding, &c. . . . . . . 412
TILLET, Dr. on the Effects of Cold Affusions in external Inflammations 122
TILLOY, M. Citric Acid from Gooseberries . . . . 366
TOWNSEND, Mr. cases illustrative of the Utility of the Stethoscope . 75
TURNER, Prof. on spontaneous Obstruction of the Canals of Arteries . 150
VULPES, M. (Naples,) on the use of Sulphate of Quinine in Fevers . 172
WACKENRODER, Mr. on Chorydalin, a new Vegetable Alkali . . 367
WALLACE, Mr. on the use of Tobacco in Hernia . . . 114
WALLER, Mr. Report of Cases in Midwifery . . . . 18
WALTON, Dr. on Organic Disease of the Heart causing Sudden Death . 135
WATSON, Mr. on the Human Eye . . . . . . 327
WEEKS, Mr. his case of Rupture of the Stomach, produced by Vomiting 265
WHITE, Mr. (America,) successful case of Ligature of the internal Iliac Artery . . . . . . . . . . . 30
WILLIAMS, Dr. on the Physical Signs of Diseases of the Lungs and Pleura, 258
WRIGHT, Dr. on utility of Pressure in Reunion of Fractured Bones . 494

J. and C. Adlard, Printers, Bartholomew Close.

### OPENING OF
### THE UNIVERSITY OF LONDON,
### FOR THE SESSION 1828—9.

THE Council published last July, a general view of the nature and objects of the Institution. They are now advanced so far in their preparations for opening the University as to be able to lay before the public an account of the proposed method of tuition and of the Courses of Study adapted to particular objects; with a detail of the days and hours when the several Professors are to teach, and of the Fees.

This "Second Statement" is to be had of J. Taylor, Bookseller to the University, 30, Upper Gower Street; Longman and Co., Paternoster Row; Murray, Albemarle Street; at the University Chambers, 29, Percy Street, Bedford Square; of Black, Edinburgh; and Hodges and Smith, Dublin. Price 1s. 6d.

The "First Statement" to be had as above, Price 1s.

The following Abstract contains the more important parts of it:

I. *Of the Days and Hours when the different Branches of Education are to be taught.*

The different branches of study for which Professors have been appointed, are such as properly belong to an University; and in the arrangement of the subjects to be taught in the Classes which will be attended by the junior students, it has been assumed that they will come possessed of that elementary knowledge which boys who leave school at fourteen or fifteen years of age have generally acquired.

The Lecture Rooms will be open to all who comply with the terms and the regulations of the University, without limitation as to age, and without examination as an indispensable preliminary. Persons who wish to attend the Lectures of one Professor only will be admitted; but those who intend to apply for University Certificates and other distinctions, must go through certain courses of study; for these testimonials will be granted to such students only, as upon examination at regular intervals are found to possess that knowledge by which the value of the Certificate or of the academical honour will be determined.

The following are the days and hours when the different Classes are to be taught. Unless otherwise expressed, the Lectures are to continue throughout the whole Academical Session, which will commence the First of November, and continue to the middle of July, with the exception of the Medical Classes, which will open the First of October and terminate about the end of May. There will be short vacations at Christmas and Easter.

ROMAN LANGUAGE. THE REV. JOHN WILLIAMS, A.M. of Balliol College, Oxford.

*Junior Class.* Every morning, (except Saturday,) from 8½ to 10½. About 340 hours of instruction, Fee 7*l.* 10*s.*—*Senior Class.* Every morning, (except Saturday,) from 11 to 12½. On Saturday, from 10½ to 12. About 300 hours of instruction, Fee 7*l.* 10*s.*

GREEK LANGUAGE. GEORGE LONG, Esq. A.M. late Fellow of Trin. Coll., Cambridge.

*Junior Class.* Every day, (except Saturday,) from 11½ to 1½. About 340 hours of instruction, Fee 7*l.* 10*s.*—*Senior Class.* Every morning, (except Saturday,) from 8½ to 10½. About 340 hours of instruction, Fee 7*l.* 10*s.*

ENGLISH LANGUAGE. THE REV. THOMAS DALE, A.M., of Corp. Chris. Coll., Camb.

Class for the Principles and Practice of Composition. Tuesday and Thursday, from 2½ to 4. Saturday, from 8½ to 10. About 150 hours of instruction, Fee 6*l.*

*English Literature.* Monday and Friday, from 1 to 2. About 70 hours of instruction, Fee 4*l.*

GERMAN LANGUAGE. LUDWIG VON MÜHLENFELS, LL.D.

Tuesday and Thursday, from 2½ to 4. Saturday Morning, from 8½ to 10. About 150 hours of instruction, Fee 6*l.*

*German Literature.* Wednesday and Saturday, from 1 to 2. About 70 hours of instruction, Fee 4*l.*

ITALIAN LANGUAGE. AN. PANIZZI, LL.D.

Monday, Wednesday, and Friday Mornings, from 10½ to 11½. About 100 hours of instruction, Fee 5*l.*

*Italian Literature.* Wednesday and Friday, from 3 to 4. About 70 hours of instruction, Fee 4*l.*

SPANISH LANGUAGE. DON A. GALIANO.

Tuesday, Thursday, and Saturday Mornings, from 10½ to 11½. About 100 hours of instruction, Fee 5*l.*

*Spanish Literature.* Tuesday and Thursday, from 3 to 4. About 70 hours of instruction, fee 4*l.*

HEBREW. HYMAN HURWITZ, Esq.

*Junior Class.* Monday, Wednesday, and Friday Mornings, from 9½ to 10½. About 100 hours of instruction, Fee 5*l* —*Senior Class.* Tuesday and Thursday Mornings, from 9½ to 10½. About 100 hours of instruction, Fee 5*l.*

HINDOOSTANEE. J. B. GILCHRIST, LL.D.

Students will have the liberty of attending the Professor of Hindoostanee for such portion of the Session as shall suit their convenience. The Fee for one hour daily during the Session will be 7*l.* 10*s.*

ORIENTAL LITERATURE. FREDERIC ROSEN, Phil. Doct.

Dr. Rosen will give instruction in the Arabic, Sanskrit, and Persian Languages, and the arrangements will be made to suit the convenience of students, as soon as the Professor shall have had a personal communication with those who wish to attend his Classes.

MATHEMATICS. AUGUSTUS DE MORGAN, Esq. A.B., of Trinity College, Cambridge.

*Junior Class.* Monday, Wednesday, Friday, from 2½ to 4. Saturday, from 10½ to 12. About 200 hours of instruction, Fee 7*l.*—*Senior Class.* Tuesday and Thursday, from 2½ to 4. Saturday, from 8½ to 10. About 150 hours of instruction, Fee 6*l.*

NATURAL PHILOSOPHY AND ASTRONOMY. The REV. D. LARDNER, LL.D., F.R.S.

*Junior Class.* Every day, (except Saturday,) from 1 to 2. About 170 hours of instruction, Fee 7*l.* —*Senior Class.* Tuesday, Thursday, and Saturday, from 11 to 12. About 100 hours of instruction, Fee 6*l.*

**CHEMISTRY.** Ed. Turner, M.D., F.R.S.E.
Every morning, (including Saturday,) from 10 to 11. *First Course.* About 100 hours of instruction, Fee 4*l.* *Second Course.* About 100 hours of instruction, Fee 3*l.* These Lectures, forming a branch of general as well as of Medical education, will commence on the First of November.

It is intended that Occasional Lectures shall be given during the Session, upon The Application of Mechanical Philosophy to the Arts, upon Civil Engineering, and upon Chemistry applied to the Arts, the particulars of which will be announced hereafter.

**BOTANY.** John Lindley, F.R.S.
Every morning, (including Saturday,) in May, June, and July, from 8 to 9. About 80 hours of instruction, Fee 4*l.*

**POLITICAL ECONOMY.** John R. MacCulloch, Esq.
Monday, Wednesday, Friday, and Saturday, from 10 to 11, from 1st of February, to the End of the Academical Session. About 100 hours of instruction, Fee 5*l.*

**JURISPRUDENCE.** John Austin, Esq., Barrister at law.
Monday, Wednesday and Friday Mornings, from 9 to 10. About 100 hours of instruction, Fee 5*l.* These Lectures will be suspended during the Quarter Sessions and Spring Circuit.

**ENGLISH LAW.** Andrew Amos, Esq., Barrister at Law, late Fellow of Trin. Coll., Camb.
Monday, Wednesday, and Friday Evenings, (except during the Quarter Sessions and Spring Circuit,) from 6½ to 7½. About 100 hours of instruction, Fee 5*l.*

**ANATOMY.** Granville Sharp Pattison, Esq.
Every day, (except Saturday,) from 2 to 3½.
*First Course* } About 120 hours of instruction each, } Fee 4*l.*
*Second Course* } 3*l.*

**DISSECTIONS AND DEMONSTRATIONS.** James R. Bennett, Esq.

*First Course* } Four Months each, } Fee 3*l.*

*Second Course* } 2*l.*

**PHYSIOLOGY.** Charles Bell, Esq., F.R.S.
Monday, Wednesday, and Friday, from 11 to 12.
*First Course* } About 50 hours of instruction each, } Fee 3*l.*
*Second Course* } 2*l.*

**COMPARATIVE ANATOMY AND ZOOLOGY.** Robert E. Grant, M.D.
Monday, Wednesday, and Friday, from 3 to 4. About 100 hours of instruction, Fee 5*l.*

**NATURE AND TREATMENT OF DISEASES.** John Conolly, M.D.
Every morning, (except Saturday,) from 9 to 10.
*First Course* } About 80 hours of instruction each, } Fee 3*l.*
*Second Course* } 3*l.*

**MIDWIFERY.** David D. Davis, M.D.
Monday, Tuesday, Thursday, and Friday, from 10 to 11.
*First Course* } About 60 hours of instruction each, } Fee 3*l.*
*Second Course* } 3*l.*

**MATERIA MEDICA AND PHARMACY.** Anthony Todd Thomson, M.D.
Every morning, (except Saturday,) from 8 to 9.
*First Course* } About 80 hours of instruction each, } Fee 3*l.*
*Second Course* } 3*l.*

*Hospital Attendance.* Aware of the great importance of affording to the pupils of a Medical School the opportunity of observing Hospital practice, and of receiving Clinical instruction, the Council will make every effort to obtain a Hospital which shall be under their own direction. In the mean time, an opportunity has occurred of making an arrangement by which the pupils will have it in their power to witness the Medical and Surgical practice in the Middlesex Hospital; and Dr. Watson, one of the Physicians, and Charles Bell, Esq. one of the Surgeons, of that Hospital, having accepted the appointments of Professors of Clinical Medicine and Clinical Surgery, the Lectures in this department will be delivered at the University as follows:

Clinical Medicine, on Monday and Wednesday Evenings, from 6 to 7.
About 60 hours of instruction, Fee £4.

Clinical Surgery, on Tuesday and Thursday Evenings, from 6 to 7.
About 60 hours of instruction, Fee £4. *

*Dispensary Attendance.* The Council are preparing to establish a Dispensary in one of the adjoining streets, to be attended by the Medical and Surgical Professors of the University.

---

The Council will continue, until further notice, to receive applications from Candidates for the following Professorships which are still vacant.

Logic and Philosophy of Human Mind. Moral and Political Philosophy. History, Ancient and Modern. Surgery. Mineralogy and Geology. French Language and Literature.

II. *Of the Fees to be paid by the Students.*

The Fee which has been stated to be payable for each Course includes the remuneration to the Professor, and a payment to the fund from which the annual expenses of the University are to be defrayed. It has been deemed necessary to state the number of hours of tuition, including examinations, because the term "*Course of Lectures*," as commonly used, would give an inadequate idea of the amount of instruction which the student will receive at the University for each Fee.

That scale of Fees applies to students who shall be nominated by a Proprietor; all who are not so nominated must pay an addition of 1*l.* 10*s.* to the University Fund for each Course of Lectures, continuing throughout the Academical Session, unless they attend more than three. If they attend a greater number, this extra payment will not be required for the additional Courses. The additional Fee to be paid by students not nominated, will be 15*s.* for each of those Courses which continue during one half of the Session, and for the Summer Course of Botany.

Those who become regular University students,—that is, students entered for a complete course of education, whether general or professional,—must pay a Matriculation Fee of 2*l.* the first year of their attendance, which will entitle them to admission to the Library and Museums for four successive Sessions. Occasional students must pay annually 10*s.* for one Course, and 1*l.* for two or more Courses, which payment will entitle them to admission to the Library and Museums.

The Council are now ready to receive the names of Pupils who mean to attend the University during the Session which is to commence next October.

The Register in which the names will be in-

---

* Until a Professor of Surgery is appointed, Mr. Bell will make such additions to this Course as shall make it sufficiently comprehensive for the Certificate required by the College of Surgeons.

scribed, will be found, until further notice, at the University Chambers, No. 29, Percy Street, Bedford Square, every day, except Sunday, from nine in the morning to five in the afternoon. The parents and friends of pupils, or the pupils themselves who may wish to see The Warden before entering their names, may call at the Chambers between Two and Five o'clock, on Mondays, Wednesdays, or Fridays. As an interview is desirable between the Professors and their pupils, in order that the Professor may ascertain the state of preparation of the student, the address of each may be obtained at the Chambers also. Students are requested to enter their names, and see the Professors whose Courses they mean to follow, as early as possible, as it is important for the interest of the students that the Professor should know their previous attainments, so that he may adapt his instruction accordingly.

Proprietors have the right of having one student of their nomination at the University for every share of One Hundred Pounds which they possess; and Donors of Fifty Pounds have the same privilege.

### III. *Of the Courses of Study.*

It is probable that, in the majority of instances, those who enter as regular University students will be disposed to attend the Lectures in that order which the Council think most advantageous for a complete general education. To such students the following Course for four years is recommended. Those who are already advanced beyond the Junior Classes may begin in that part of the Course for which they are most fitted by previous attainments; but as the examinations for the University Certificates and other distinctions will include what is taught in the earlier as well as in the later years of study, the pupil ought to ascertain before he passes over a Junior Class, that he is fully prepared to undergo an examination upon all that is to be learned in it.

First Year.—Junior Latin, Junior Greek, and Junior Mathematics.

Second Year.—Senior Latin, Senior Greek, and Senior Mathematics.

A student may this year substitute *Junior Natural Philosophy* for *Senior Mathematics*, or he may attend both Classes.

Third Year. — Chemistry, Junior Natural Philosophy, Logic, and the Philosophy of the Human Mind.

Fourth Year.—Jurisprudence, Political Economy, Senior Natural Philosophy, and Moral and Political Philosophy.

| | £. | s. | d. |
|---|---|---|---|
| Average annual expense of the four years to students nominated by a Proprietor for the above education | 22 | 7 | 6 |
| Average annual expense of the four years to students not nominated . | 26 | 17 | 6 |

In addition to the above branches, the student will have time to study the Modern Languages and attend the Lectures on History, and some of the most popular among the Professional Courses.

The arrangements for the Medical School have been so framed as to enable the pupils to comply with the regulations which must be observed by Candidates for the Diplomas of the Royal College of Surgeons, or the Company of Apothecaries.

The expense of the Courses required by the College of Surgeons to a student at the University, will be as follows:

| | £. | s. | d. |
|---|---|---|---|
| 3 Courses of Anatomy . . . . . . | 10 | 0 | 0 |
| 1 Course of Physiology . . . . | 3 | 0 | 0 |
| 1 Course of Surgery . . . . . . . | 3 | 0 | 0 |
| 1 Course of Chemistry . . . . . . | 4 | 0 | 0 |
| 2 Courses of Midwifery . . . . | 6 | 0 | 0 |
| Dissections and Demonstrations . . | 5 | 0 | 0 |
| Matriculation Fee . . . . . . . | 2 | 0 | 0 |
| Total Fees of the University, if nominated by a Proprietor . . . | 33 | 0 | 0 |
| Addition, if not nominated . . . | 4 | 10 | 0 |
| | 37 | 10 | 0 |
| One Year at Middlesex Hospital . . | 22 | 6 | 0 |
| | 59 | 16 | 0 |

The expense of the Courses required by the Company of Apothecaries to a student at the University, will be as follows:

| | £. | s. | d. |
|---|---|---|---|
| 1 Course of Materia Medica . . . | 3 | 0 | 0 |
| 1 Course of Chemistry . . . . . . | 4 | 0 | 0 |
| 2 Courses of Anatomy . . . . . . | 7 | 0 | 0 |
| 1 Course of Physiology . . . . . | 3 | 0 | 0 |
| 2 Courses of Practice of Medicine . | 6 | 0 | 0 |
| Matriculation Fee . . . . . . . . | 2 | 0 | 0 |
| Total Fees of the University, if nominated by a Proprietor . . . | 25 | 0 | 0 |
| Addition, if not nominated . . . . . | 4 | 10 | 0 |
| | 29 | 10 | 0 |
| Six Months at Middlesex Hospital . | 10 | 10 | 0 |
| | 40 | 0 | 0 |
| Or, Nine Months at the Dispensary . | 7 | 7 | 0 |
| Fees as above . . . . . . . . . | 29 | 10 | 0 |
| | 36 | 17 | 0 |

The time which the Professors are to devote to their pupils will be considerably greater, in most of the Classes nearly one half more, than has hitherto been the case in the Courses delivered at the Medical Schools in London; for the subjects of the Lectures will be treated more minutely, and there will also be weekly examinations of the pupils.

Rooms are provided for the accommodation of the pupils during the intervals of lecture; and suitable refreshments will be sold within the Building, by a Steward (to be appointed by the Council) upon his own account, but subject to regulations both as to what he is to supply and the prices to be charged.

### IV. *Of Examinations and Certificates.*

It is intended that *in every class* in the Uni versity, the Professor shall devote a certain por tion of the hours of instruction in each week t the examination of his pupils.

The manner of conducting these examination and the frequency of their recurrence, must ne cessarily vary; and as each Professorship wil probably require a separate plan of examinatio the details can only be properly settled in concer with the several Professors.

Persons who may be desirous of attendin Lectures in the University, without submittin to examination, will be at liberty to do so. N student, however, who wishes to obtain a Certif cate can be exempted from the examinations.

It is intended that, in addition to the week examinations, there shall be at the conclusion every Session a public examination of all wh may be desirous of obtaining a Certificate from the Professor whose Course they have attended. As the value of these documents must depend upon the strictness of the examinations, such a system will be adopted as shall most accurately determine the attainments of those to whom they are granted.

Besides Certificates of the Professors, the University will grant Certificates of general proficiency in literature and science. Every student will be required to produce a certain number of Professors' Certificates, before he can be allowed to enter upon the examination for the General Certificate.

It would be premature at present to fix the conditions on which General Certificates will be granted. The Council will be enabled hereafter to frame the regulations with greater precision, profiting by the suggestions which experience will afford, when the system of the University is in operation.

### V. *Library and Museums.*

The Council have set apart a portion of the funds at their disposal for collections in Anatomy, Natural History, Books, and Philosophical apparatus; and they propose in the month of October to open the small Library and Anatomical Museum. The Council have availed themselves of an opportunity of adding to this Museum a more perfect collection of drawings of morbid structure than, it is believed, has hitherto been applied to the purposes of teaching and study, and which will be peculiarly valuable to the student of the Practice of Medicine. Dr. A. T. Thomson is :ollecting a Museum of Materia Medica on a nore extensive scale than has hitherto been atempted in that branch of medical science.

Dr. Lardner has been employed nearly a year n the collection of philosophical apparatus; and Dr. Turner is preparing all that is necessary to ender the laboratory complete and efficient.

### VI. *Houses for the Reception of Boarders.*

The Council feel that their direct interference n the management of houses opened for the reeption of boarders must necessarily be inefcient; and unwilling to give a pledge which hey cannot redeem, they will not attempt to lay own any rules for the conduct of the students eyond the walls of the University. They there›re earnestly recommend to parents and guarians, in the first instance, to be scrupulously areful in examining the references given by ersons opening such houses, and the experience f a very short period will establish the character f those in whom confidence may be placed.

Some of the Professors have informed the Varden that they intend to receive boarders in ieir families.

---

This Statement by the Council is followed by brief Outline by each Professor, pointing out e manner in which they mean to treat their veral subjects.

---

### ADVERTISEMENTS.

### RELIGIOUS INSTRUCTION.

'E, the undersigned Professors in the University London, who are Clergymen of the Estaished Church, having from the period of our pointment entertained the intention of providing religious instruction for those students who are members of our Church, do hereby Give Notice, That final arrangements have been at length made, with the full approbation of the Council, for that purpose.

An Episcopal Chapel has been purchased contiguous to the University, to be called "The University Chapel," where accommodation will be afforded to the students for attendance at divine service, and where a Course of Divinity Lectures will be regularly delivered during the Academical Session. Parents and others interested in this arrangement, may learn further particulars by applying to Mr. Taylor, 30, Upper Gower Street.

Thomas Dale, M.A. Camb.
Dionysius Lardner, LL.D. Dublin.
John Williams, M.A. Oxon.

---

### BOARDING HOUSES.

Mr. Taylor, Bookseller and Publisher to the University, No. 30, Upper Gower Street, with the sanction of the Council, has opened a Register, in which the names of those Housekeepers will be inserted who are willing to receive Boarders. Those who wish to have their names entered and retained in this Register, must comply with the following Conditions:

That the persons applying shall produce satisfactory proof of correctness of character, with regard to religious and moral habits. A testimonial from the Minister to whose congregation they belong, will be indispensable.

That they will require their boarders to be at home at an early hour of the night.

That they will not suffer gaming or licentious conduct on the part of the boarders.

That they will require their boarders to attend some place of public worship.

That they will make an immediate report to the parents or friends of their boarders, in case of any irregularity of conduct or serious illness.

That they will not receive any boarders except students of the University.

The names of such persons as shall be discovered to have evaded these rules, will be erased from the Register, and notice of that erasure will be sent to the parents or relatives of all the students who may be boarded in their houses.

*** Notice must be sent to Mr. Taylor of the names of the Boarders, and the names and addresses of their parents or relatives.

THE REGISTER WILL CONTAIN,

The names and addresses of the applicants.
Their religious persuasion.
The names and addresses of the persons by whom they are recommended.
The description of the several persons of whom their family consists.
The number of boarders they propose to take.
Their terms.

A Fee of Five Shillings must be paid for Registration, and an additional Fee of Five Shillings annually for each Boarder.

### PRIVATE TUTORS.

Mr. Taylor, with the sanction of the Council, has also opened a Register, for receiving the names of Private Tutors, in the various Departments of Literature and Science taught at the University.

---

Printed by Richard Taylor, Red Lion Court, Fleet Street.

